# A resonant high-pressure microsensor based on a composite pressure-sensitive mechanism of diaphragm bending and volume compression

**DOI:** 10.1038/s41378-024-00667-8

**Published:** 2024-03-15

**Authors:** Pan Qian, Zongze Yu, Jie Yu, Yulan Lu, Bo Xie, Jian Chen, Deyong Chen, Junbo Wang

**Affiliations:** 1 The State Key Laboratory of Transducer Technology, Aerospace Information Research Institute, Chinese Academy of Sciences, Beijing, 100190 China; 2 The School of Electronic, Electrical and Communication Engineering, University of Chinese Academy of Sciences, Beijing, 100049 China

**Keywords:** Electrical and electronic engineering, Engineering

## Abstract

In this paper, a composite pressure-sensitive mechanism combining diaphragm bending and volume compression was developed for resonant pressure microsensors to achieve high-pressure measurements with excellent accuracy. The composite mechanism was explained, and the sensor structure was designed based on theoretical analysis and finite element simulation. An all-silicon resonant high-pressure microsensor with multiple miniaturized cavities and dual resonators was developed, where dual resonators positioned in two resonant cavities with suitably different widths are used to perform opposite characteristics in pressure and the same characteristics at different temperatures, which can improve pressure sensitivities and realize temperature self-compensation by differential frequency output. The microsensor was fabricated by microfabrication, and the experimental results showed that the sensor had an accuracy of ±0.015% full scale (FS) in a pressure range of 0.1~100 MPa and a temperature range of −10~50 °C. The pressure sensitivity of the differential frequency was 261.10 Hz/MPa (~2523 ppm/MPa) at a temperature of 20 °C, and the temperature sensitivities of the dual resonators were −1.54 Hz/°C (~−14.5 ppm/°C) and −1.57 Hz/°C (~−15.6 ppm/°C) at a pressure of 2 MPa. The differential output had an outstanding stability within ±0.02 Hz under constant temperature and pressure. Thus, this research provides a convenient solution for high-pressure measurements because of its advantages, namely, large range, excellent accuracy and stability.

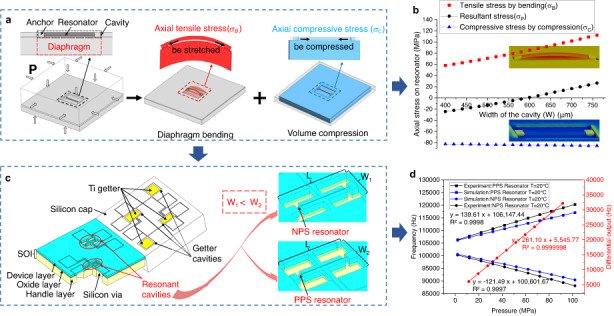

## Introduction

Microelectromechanical system (MEMS) pressure sensors, which are widely used in aerospace, biomedicine, industrial control and environmental monitoring, have the advantages of low power consumption, small size, low cost and small impact on measured objects^1–3^. However, high-pressure and high-accuracy pressure microsensors are urgently needed to meet the needs of high-pressure detection in harsh environments, such as downhole drilling and oil extraction, gas compression and transportation, ocean exploration and disaster forecasting^4–6^. In several studies, piezoresistive or capacitive MEMS pressure sensors have been used to achieve high-pressure measurements^7–10^, and a few piezoresistive pressure microsensors have utilized microcavities instead of pressure-sensitive diaphragms to form a stress concentration area by introducing volumetric compression effects^11–13^, easily expanding the pressure range even to GPa. However, these piezoresistive and capacitive high-pressure microsensors all lacked full-range accuracy because of severe temperature disturbances or poor linearity.

At present, in many studies, silicon resonant pressure microsensors are employed to achieve high-pressure measurements, demonstrating great advantages in terms of accuracy, stability and anti-interference ability owing to quasidigital outputs^14^. Most resonant high-pressure microsensors are constructed with diaphragm-sensitive structures for pressure/stress conversions. For instance, J. C. Greenwood^15^ developed a silicon resonant high-pressure microsensor with a pressure range up to 7000 psi (~48.3 MPa) based on a thick diaphragm, but the off-chip temperature sensor exhibited hysteresis. Lu et al.^16^ reported a resonant high-pressure microsensor with effective temperature self-compensation enabled by dual resonators, which were located in zones of tensile and compressive stresses of a square diaphragm, with an accuracy of ±0.01% full scale (FS) in the range of 0.02~1 MPa. Later, Xiang et al.^17^ and Yu et al.^18^ extended the pressure range to 7 MPa and 30 MPa, respectively, by further optimizing the geometrical parameters of the diaphragm structure, which retained high accuracy; however, their diaphragms were no longer strong enough to withstand higher pressures. To achieve a high range, increasing the thickness or decreasing the area of the diaphragm can stiffen the structure, while the stress on the diaphragm will decrease considerably on a par with the volume compression stress^15,19,20^. Therefore, higher pressure microsensors cannot be generated by relying only on the diaphragm for pressure/stress conversions.

Volume compression of monocrystalline silicon for pressure/stress conversions has been used to design resonant high-pressure sensors with large working ranges. To avoid buckling during high-pressure measurements, Ryuuichirou Noda and Toshiki Mitsuhashi et al. developed a groove diffusion method to apply residual tensile strains on a resonator, and a newly developed resonant high-pressure sensor was used to measure a pressure range of 70 MPa^20,21^. However, high-pressure sensors based only on volume compression cannot achieve self-compensation due to the absence of differential dual resonators with opposite pressure characteristics, leading to compromised accuracy in high-pressure measurements. The pressure sensitivity is certain, limited by the volume compression rate, and even low linearity is shown in ultrahigh-pressure measurements.

To solve the aforementioned problems of resonant high-pressure sensors, this paper developed a composite pressure-sensitive mechanism combining diaphragm bending and volume compression to realize pressure/stress transitions. In addition, a multicavity all-silicon resonant pressure microsensor with dual resonators was designed and fabricated to achieve high-accuracy measurements at high pressures. Multiple miniaturized cavities were adopted to improve the pressure resistance and ensure the deposition of enough getter materials for high vacuum. Resonators positioned in cavities with different widths can match positive and negative pressure sensitivities and temperature self-compensations. In this study, the stress and frequency characteristics were analyzed based on finite element simulations, and an isolation layer of silicon stresses was included according to thermal stress analysis to realize a low-stress assembly. An all-silicon microsensor with low temperature disturbances in an oil-filled isolation structure was used to test actual high pressures with verified excellent results.

## Materials and methods

### Structure and working principle

**Fig. 1:**
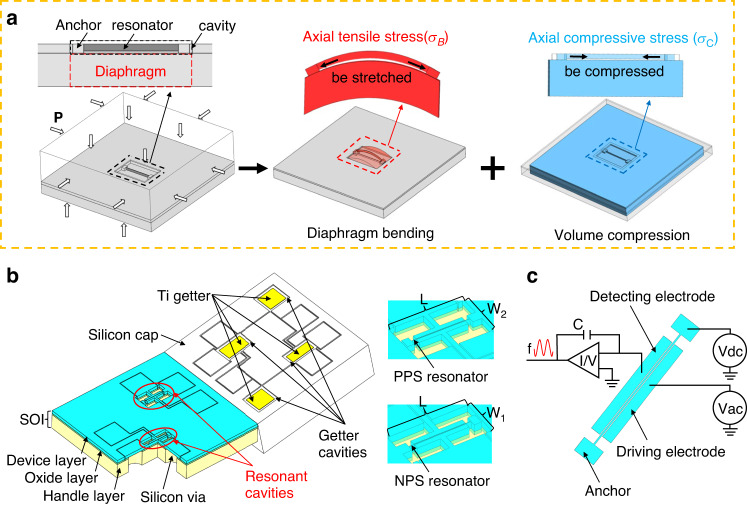
The overall design of the resonant high-pressure microsensor. **a** composite pressure-sensitive mechanism of diaphragm bending and volume compression; **b** schematic of multicavity all-silicon resonant high-pressure microsensor with dual resonators; and **c** excitation and pickup of a resonator

The stress state of the resonator anchored on the bottom surface of a cavity can reflect the external pressure via a composite mechanism, as shown in Fig. 1a. The cavity containing resonators can construct a composite structure with diaphragm bending and volume compression; thus, the stress changes in the resonant beam result from these two mechanisms when all the outer surfaces of the block are subjected to pressures during measurements. On the one hand, the anchoring plane of the beam corresponding to the cavity is the pressure-sensitive diaphragm, and the beam can be stretched by diaphragm bending to exhibit a tensile axial stress (positive stress *σ*_*B*_). On the other hand, the beam shows a compressive axial stress (negative stress *σ*_*C*_) only due to the effect of volume compression. The resultant axial stress (*σ*_*P*_) caused by the total external pressure on the beam is shown in Eq. (1):

1 $${\sigma }_{P}={\sigma }_{B}+{\sigma }_{C}$$

To achieve higher pressure resistance, we chose a rectangular diaphragm, which fits better with long beam structures and facilitates the miniaturization of cavities. More importantly, rectangular diaphragms are more conducive to the design of dual resonators for self-compensation and are adopted to achieve high accuracy. The compression deformation of the body is mainly determined by the volume compression rate of the material, and the deformation of the sensitive diaphragm is mainly affected by the size of the area. For a determined resonator size and cavity length, the width of the cavity (W) can be changed to achieve the resultant stress on the beam in different states. Therefore, dual resonators with opposite pressure characteristics and the same temperature characteristics can be easily achieved by varying the width of the rectangular diaphragms. Dual resonators for measuring both pressure and temperature can realize self-compensation with better performance than other temperature compensation methods that rely on cantilever resonators or temperature sensors^22–25^. This is because there are noise stresses from other factors, such as packaging, in traditional temperature compensation approaches.

Through this composite mechanism, a new resonant high-pressure microsensor was developed whose miniaturized cavities stiffened the diaphragm structure to achieve a much greater range. Additionally, cavities with dual resonators of different widths were utilized to achieve high accuracy. Figure 1b shows an all-silicon resonant high-pressure microsensor with dual resonators and multiple small cavities. The microsensor was composed of a silicon on insulator (SOI) wafer and a silicon cap, in which the core components were resonators placed in two resonant cavities with different widths on the SOI. Under the condition of W2 > W1, the resonator beams demonstrated opposite pressure/stress conversions. A single-beam resonator was chosen, which was anchored in the central position of the diaphragm, leading to a uniform stress distribution. For a single-beam resonator with transverse vibration, the intrinsic resonant frequency *f*_0_ is shown in Eq. (2):

2 $${f}_{0}=1.028\sqrt{\frac{E}{\rho }}\frac{b}{{l}^{2}}$$

where *b* is the resonator width, *l* is the resonator length, *ρ* is the silicon density, and *E* is the Young’s modulus related to temperature. When the resonant beam is subjected to axial stress, the frequency (*f*_*σ*_) of the resonator is related to the total stress (*σ*) on the beam, as shown in Eq. (3):

3 $${f}_{\sigma }={f}_{0}\sqrt{1+\frac{\sigma }{{\sigma }_{E}}}$$

If σ is a small stress, then *f*_*σ*_ can be approximated by a linear relationship with *σ*, as shown in Eq. (4):

4 $${f}_{\sigma }={f}_{0}\left(1+\frac{\sigma }{2{\sigma }_{E}}\right)={f}_{0}(1+\frac{{\sigma }_{P}+{\sigma }_{T}}{2{\sigma }_{E}})$$

where *σ*_*P*_ is the resultant stress by pressure, *σ*_*T*_ is the noise stress, which mainly includes thermal stresses, and *σ*_*E*_ is the critical Euler stress related to temperature. Noise stress (*σ*_*T*_) can be ignored in all-silicon sensors with two identical resonators sensing both pressure and temperature.

The relationship between linearity and the full-scale frequency variation was analyzed, as shown in Supplementary Fig. S1. When the frequency variation is approximately 10% of the fundamental frequency where the total stress (*σ*) is approximately 0.2*σ*_*E*_, both the sensitivity and linearity are excellent. The ideal tensile or compressive stress can be achieved by adjusting and matching the relative magnitude of diaphragm bending and volume compression. The resonator placed in the wider resonant cavity had a positive pressure sensitivity (PPS resonator with *f*_*PPS*_), and the counterpart in the narrower resonant cavity had a matched negative pressure sensitivity (NPS resonator with *f*_*NPS*_); however, they had numerically identical *σ*_*P*_ and the same temperature characteristics. Thus, dual resonators can improve the pressure sensitivity by differential measurements and realize temperature self-compensation. The principle of accurate measurements is shown in Eq. (5):

5 $$\begin{array}{lll}\left\{\begin{array}{l}{f}_{{PPS}}-{f}_{{NPS}}=\left({f}_{0}\frac{{\sigma }_{P}}{{\sigma }_{E}}\right)={F}_{1}\left(\,P,T\,\right)\\ {f}_{{PPS}}+{f}_{{NPS}}=2{f}_{0}={F}_{2}\left(\,T\,\right)\end{array}\right. & \to & P=F({f}_{{PPS}},{f}_{{NPS}})\end{array}$$

A silicon cap was used for vacuum packaging in this study to reduce temperature disturbance. Since a sufficient amount of getter was required to maintain quality^26^, several small getter cavities on the silicon cap were used to ensure a high vacuum. Electrostatic excitation and capacitance detection were adopted to ensure compatibility with high-pressure environments, while the excitation and pickup of the resonator are shown in Fig. 1c.

### Stress analysis and assembly design

For high resistance, the size of the resonant beam should be as small as possible due to the geometrical limitations of the cavities. When the feasibility of fabrication and stability of testing were considered, the resonant beam size was determined to be 10 μm in width and 850 μm in length with a 100 kHz resonant frequency, while the length of the cavity was determined as 1100 μm. Since the actual stress state was very complicated, the simulated normal stress along the beam axis was used as a reference for theoretical analysis. The dimensions not specified below are width × length × height.

**Fig. 2:**
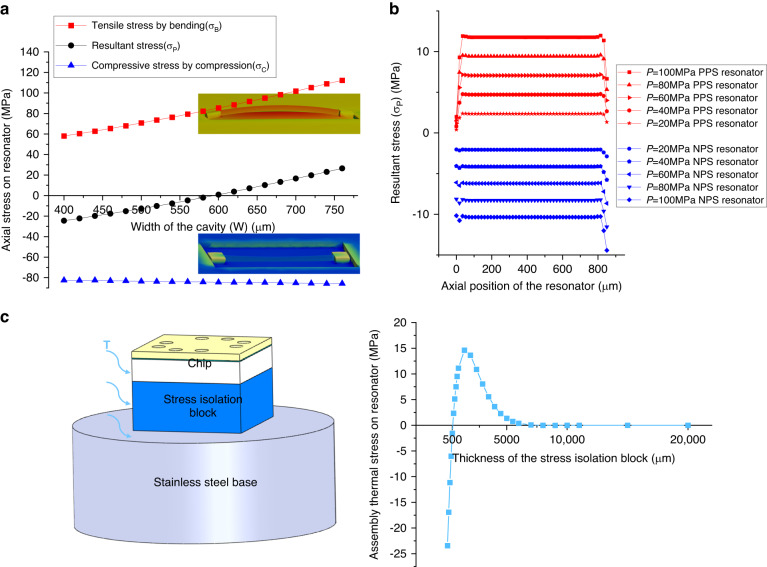
Results of the simulated stress characteristics and stress isolation structure. **a** axial stresses on the resonator in cavity of different widths; **b** resultant axial stresses on dual resonators in the sensor under different pressures; and **c** thermal stress analysis on the resonator after assembly with isolation blocks of varied thickness

For a silicon block (4 × 4 × 1.342 mm) containing a cavity (*W* × 1100 × 60 μm) with a beam, when all the outer surfaces of the block were subjected to a certain pressure of 100 MPa, the simulation model was simply divided into two upper and lower surfaces and four surrounding surfaces. When the upper and lower surfaces were under stress, the diaphragm was mainly bent, causing the tensile axial stress in the beam to increase rapidly with increasing cavity width. When the four surrounding surfaces were stressed, the volume compression mainly caused the beam to be compressed with compressive axial stress, but its magnitude was basically unaffected by the cavity width. Therefore, the resultant stress showed different states when the cavity was of different widths, changing from compressive to tensile stresses with W changing from 400 μm to 760 μm, as shown in Fig. 2a. Since the stress on the beam changes gradually with the width of the rectangular diaphragm, the magnitude of the resultant axial stress at full scale can be well controlled at approximately 0.2*σ*_*E*_. Additionally, varying the diaphragm width to achieve differential dual resonator design has excellent controllability and process tolerance, which is not possible with other geometries, such as square/circular.

Therefore, two rectangular cavities of different widths were appropriately selected for opposite pressure sensitivities between a PPS resonator (10 × 850 × 40 μm) and an NPS resonator (9.5 × 850 × 40 μm) to avoid frequency crossing. By integrating two resonators into a pressure sensor (5 × 5 × 1.342 mm) with two resonant cavities differing only in width, performance matching was realized. For the dual resonant beams ($b={10}\,\upmu m/{9.5}\,\upmu m,l=850\,\upmu m$), the Euler stress ${\sigma }_{E}=\frac{{\pi }^{2}E}{3}{(\frac{b}{l})}^{2}$ is approximately 76 MPa/68 MPa, so we choose two suitable widths to produce approximately 15 MPa of tensile stress in the PPS resonator and approximately −13 MPa of compressive stress in the NPS resonator at full scale, as shown in Fig. 2b. At this time, the dual resonators had full-scale frequency variations of ±10% fundamental frequencies, exhibiting both high sensitivity and linearity. Additionally, the beam stresses in the rectangular diaphragm were basically the same everywhere, which further improved the fabrication tolerances and realized frequency matching. This high-pressure sensor adopting a rectangular diaphragm is more conducive to achieving high accuracy, as discussed in Supplementary Fig. S2.

The sensor needed to be assembled for stable measurement under a high-pressure environment, but the assembly may introduce thermal stresses as serious and uncontrollable noise. When the external temperature changes, the large difference between the thermal expansion coefficient of silicon and stainless steel can produce large strain noise and worsen the temperature characteristics, urgently requiring a stress isolation layer to address this issue. The most commonly reported isolation structures include large profiled substrates or thick isolation blocks^27,28^. Specifically, isolation blocks were feasible for high pressures, although based on simulations of thermal stresses, the corresponding thickness needed to be greater than 5 mm to ensure a certain isolation effect when the temperature was changed from 20 °C to −50 °C, as shown in Fig. 2c. This thickness was too large for fabrication, assembly and testing. The isolation blocks were specially structured to both meet the requirements of assembly stability and machining feasibility through stress analysis, and the total thickness of the isolation layer was reduced to 2 mm after simple optimization. All the dimensions of the sensor were determined via simulation after assembly, which involved two resonant cavities with widths of 710 μm and 542 μm.

### Fabrication process

Based on the size parameters used in the structural design, material selection was achieved by means of a 4-inch SOI (a device layer of 40 μm, an oxide layer of 2 μm, and a handle layer of 300 μm) and two 4-inch silicon wafers (thicknesses of 1 mm and 2 mm). To avoid introducing other thermal stresses and achieve stable thermal stress isolation, the isolation layer material is N-type silicon with a low doping level and <100> orientation. The main fabrication processes included deep reactive ion etching (DRIE), resonator release, physical vapor deposition (PVD), and wafer bonding. More specifically, for wafer bonding, many studies have used eutectic bonding of metal and silicon for vacuum packaging^29,30^, producing all-silicon devices with excellent performance.

**Fig. 3:**
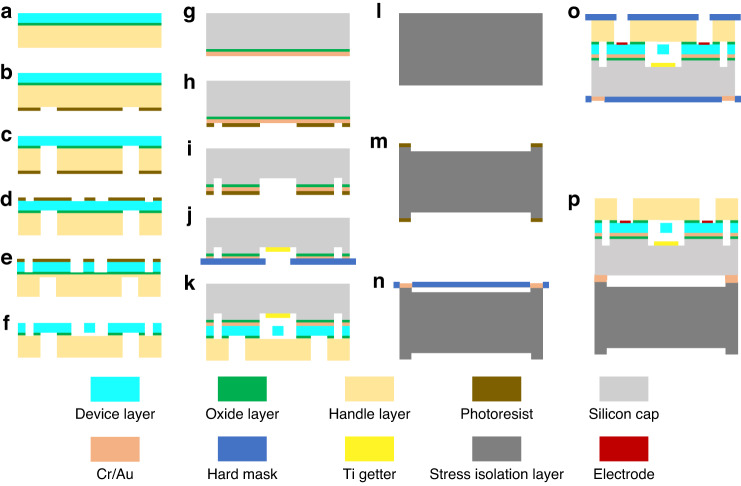
Fabrication process of the resonant high-pressure microsensor. **a** cleaning SOI; **b** first lithography; **c** etching to form silicon vias; **d** second lithography; **e** etching to form resonators; **f** releasing resonators; **g** depositing oxide layer and Cr/Au; **h** third lithography; **i** etching to form cavities; **j** depositing getter materials based on a hard mask; **k** vacuum packaging by Au-Si eutectic bonding; **l** cleaning silicon wafer; **m** fourth lithography and DRIE to form an isolation layer; **n** depositing Cr/Au on the isolation layer; **o** depositing Cr/Au and Al electrodes on the chip; and **p** assembling by AU-AU hot pressing bonding

The fabrication process of the developed sensor is shown in Fig. 3. After cleaning the SOI wafer, resonators on the device layer and silicon vias through the handle layer and the oxide layer were fabricated, as shown in Fig. 3a–e. Then, a hydrofluoric (HF) solution was used to release resonators, ensuring that the resonant beams could vibrate, as shown in Fig. 3f. For the fabrication of silicon caps, silicon dioxide and Cr/Au were first deposited on silicon wafers by low-pressure chemical vapor deposition (LPCVD) and evaporative PVD, respectively (see Fig. 3g). All the cavities were fabricated by lithography, metal corrosion, and DRIE, as shown in Fig. 3h, i. A hard mask was used to deposit the Ti getter in all the cavities, as shown in Fig. 3j. Subsequently, single chips were fabricated by Au-Si eutectic wafer bonding, as shown in Fig. 3k. The stress isolation layer mainly adopted DRIE to form special structures, and Cr/Au was deposited on four convex tables, as shown in Fig. 3l–n. Al was deposited in silicon vias, and Cr/Au was deposited on the surface of the chips, as shown in Fig. 3o. Finally, the sensor chips with stress isolation structures fabricated by Au-Au hot-pressing wafer bonding are shown in Fig. 3p.

## Results and discussion

### Physical and oil seals

**Fig. 4:**
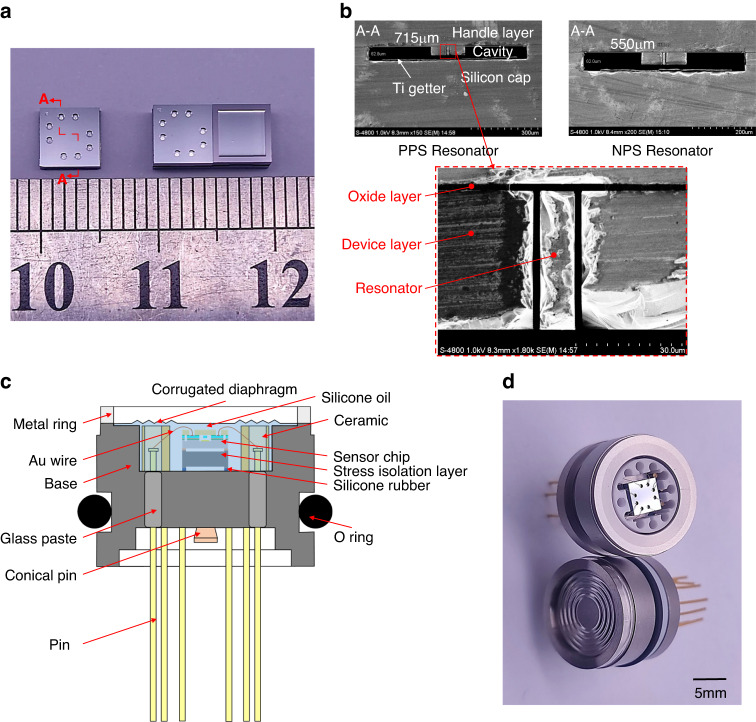
Fabricated sensor chip and oil-filled isolation package. **a** Images of the single chip and chips with stress isolation; **b** cross sections of two resonators enabled by SEM; **c** schematic of the oil-filled isolation package; and **d** high-pressure microsensor before and after oil seal

The fabricated single chip and chips with a stress isolation layer are shown in Fig. 4a, in which the total size is 5 × 5 × 3.342 mm. SEM images of the resonant cavities are shown in Fig. 4b, which indicate obvious differences in width between the two resonant cavities (*W*_1_ = 550 μm, *W*_2_ = 715 μm) and the microstructures of the resonators located in the centers of the cavities. To prevent the sensor chip from being damaged by the complex environment, the chip needs an isolated package, where a well-established oil-filled isolation package of corrugated steel diaphragm was adopted^31,32^. Figure 4c shows the cross section of the pressure sensor after packaging. The sensor chip with the stress isolation structure was adhered to a base of stainless steel by silicone rubber for low-stress assembly and then enclosed in a silicone oil-filled chamber by a corrugated diaphragm, which was isolated from the tested media to adapt to harsh environments. The high-pressure sensors before and after the oil seal are shown in Fig. 4d.

### Performance characteristics

**Fig. 5:**
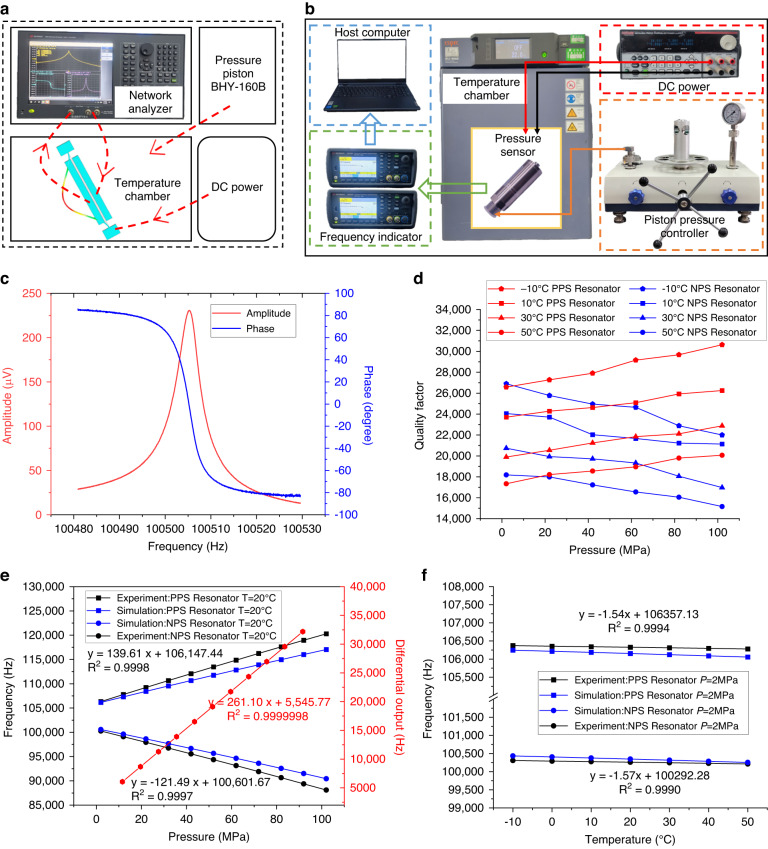
Experimental platform and test results of resonant high-pressure microsensor. **a** schematic of open-loop test platform; **b** closed-loop test experiment system; **c** resonant response at atmospheric pressure and room temperature; **d** quality factors of dual resonators under different temperatures and pressures; **e** experimental and simulated pressure sensitivities and **f** experimental and simulated temperature sensitivities

To verify the performance of the resonant pressure microsensor, open-loop and closed-loop tests were carried out (see Fig. 5a, b). A network analyzer (Keysight E5061B) was used to quantify the frequency response characteristics of the sensor by picking up and analyzing the output voltage signals of the microsensor. The temperature box (Su-262) provided different temperature environments, and the pressure source was a pressure piston (BHY-160B) with an accuracy of ±0.005% FS. A Keysight 53230 A frequency meter was used to collect the closed-loop vibration frequency of the dual resonators.

As shown in Fig. 5c, the operating frequency of the resonant pressure sensor was 100505 Hz, and the amplitude of the output voltage was approximately 230 μ*V* at room temperature and atmospheric pressure. The steep amplitude‒frequency curve can provide a large phase margin for the frequency closed loop. The quality factors of the microsensor in the measurement ranges of −10~50 °C and 0~100 MPa are shown in Fig. 5d; these values are all greater than 15000, confirming the excellent vacuum conditions. Figure 5e shows the pressure sensitivity of the dual resonators at a temperature of 20 °C, where the experimental results were basically consistent with the simulation results. The slightly larger sensitivity was mainly caused by fabrication errors. More specifically, the pressure sensitivities were quantified as 139.61 Hz/MPa (~1316 ppm/MPa) for the PPS resonator, −121.49 Hz/MPa (~−1207 ppm/MPa) for the NPS resonator and 261.10 Hz/MPa (~2523 ppm/MPa) for the differential output. Due to the all-silicon design of the sensor with low temperature disturbances, the experimental temperature sensitivities of the dual resonators were quantified as −1.54 Hz/°C (~−14.5 ppm/°C) and −1.57 Hz/°C (~−15.6 ppm/°C) (see Fig. 5f), which deviated from the simulation results because of the multistep packaging. However, the matched dual resonators exhibited the same variation in package noise stress, so they could still obtain an excellent match, resulting in better temperature self-compensation.

**Fig. 6:**
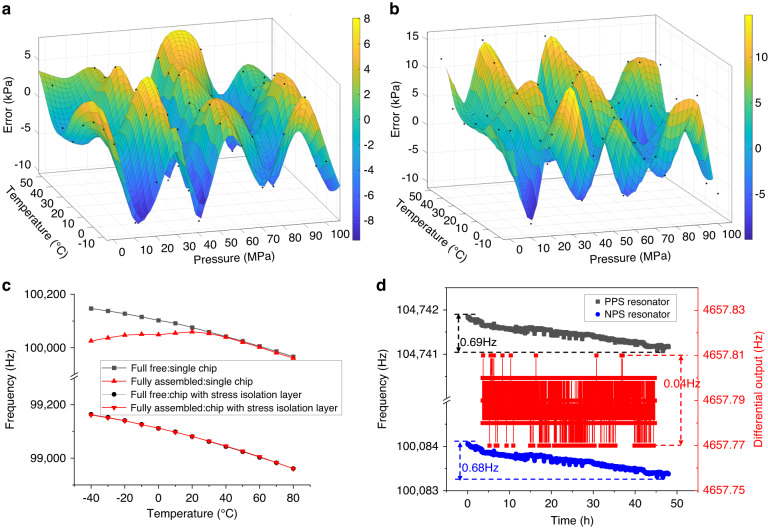
Performance characterization of high-pressure microsensor. **a** full range fitting errors; **b** full range measurement errors; **c** chip temperature sensitivities of −40~80 °C whether to assemble and isolate; and **d** stability of resonant frequency under constant temperature and pressure

In the pressure range of 0.1~100 MPa and the temperature range of −10~50 °C, the fitting errors of the sensor were obtained by a polynomial algorithm within ±10 kPa (with a fitting accuracy better than ±0.01% FS), as shown in Fig. 6a. The measurement errors of the sensor within ±15 kPa (i.e., a measurement accuracy better than ±0.015% FS) are shown in Fig. 6b. To verify the effect of the stress isolation layer, the temperature sensitivities of the single chip and the isolated chip both before and after assembly were tested at a temperature range of −40~80 °C and a pressure of 100 kPa. As shown in Fig. 6c, the temperature characteristics of the single chip changed greatly before and after assembly, even reaching an inflection point. The temperature characteristics of the assembled chip with isolation exhibited the same trend before and after assembly, indicating that the assembly had an excellent ability to address thermal stresses. Afterward, the assembled device was tested for short-term stability, as shown in Fig. 6e. Due to insufficient high-pressure aging of the sensor, the operating frequencies of the two resonators shifted in the same direction by 0.69 Hz (−0.00033% FS/day) and 0.68 Hz (−0.00034% FS/day) within 48 h at a temperature of 20 °C and a pressure of 100 kPa. However, the differential frequencies remained stable, and the interval of frequency fluctuation was within ±0.02 Hz. The importance of temperature self-compensation for dual resonators was validated.

The aforementioned results showed that the prepared sensor achieved high-accuracy measurements within a wide temperature zone and a 100 MPa pressure range, indicating high-pressure sensitivity, low temperature disturbance and dual-resonator characteristic matching. Differential output further improved the pressure sensitivity and linearity, achieved temperature self-compensation and exhibited excellent stability. A performance comparison between the results of this study and those of MEMS high-pressure sensors reported in recent years is shown in Table 1. Compared with previous studies, this study developed a novel resonant high-pressure sensor based on the composite pressure sensitivity mechanism, which demonstrated high performance in terms of key parameters such as measurement range, sensitivity and accuracy.

**Table 1:** Performance comparison of MEMS high-pressure sensors

	Principle	Measurement range	Temperature	Sensitivity	Measurement accuracy
^6^	Piezoresistive	0~120 MPa	-	~0.425 mV/V/MPa	±0.0182% FS
^33^	Piezoresistive	0~70 MPa	−55~125 °C	~143 μV/V/MPa	±0.04% FS
^15^	Resonant	0.11~30 MPa	−10~60 °C	~3461 ppm/MPa	±0.01% FS
^17^	Resonant	0~70 MPa	-	~2037 ppm/MPA	-
This work	Resonant	0.1~100 MPa	−10~50 °C	~2523 ppm/MPa	±0.015% FS

## Conclusion

In this paper, the composite pressure-sensitive mechanism of a resonant pressure microsensor was demonstrated by combining diaphragm bending and volume compression to realize pressure/stress conversions effectively, and a multicavity all-silicon resonant high-pressure microsensor with dual resonators was developed. The composite pressure-sensitive mechanism can achieve high range and high accuracy in a wide temperature region compared with two traditional single mechanisms. The matching design of dual resonators with positive and negative pressure sensitivities can easily be realized through the adaptation and combination of two single mechanisms. The differential output was shown to further improve the sensitivity and realize temperature self-compensation. Vacuum packaging and low-stress assembly of all-silicon structures were realized by gold-silicon eutectic bonding and gold-gold hot-pressing bonding, respectively, resulting in low-temperature disturbances. The experimental results validated the high performance of this microsensor in terms of accuracy, quality factor, sensitivity and stability. The design and production process also exhibit the advantages of high stress controllability and high process tolerance. However, the diaphragm of microsensors based on a composite pressure-sensitive mechanism still has a weak structure, limiting further expansion of the pressure range. To ensure high vacuum, chip miniaturization and high-yield bonding also face challenges due to the design of multiple getter cavities, which need to be optimized. Future work may focus on further optimization of the isolated assembly in terms of stress and aging of the sensor to improve the frequency stability of each resonator, with real applications in high-pressure measurements.

### Supplementary information

Supplemental Material
