# Supplementary material for: A resonant high-pressure microsensor based on a composite pressure-sensitive mechanism of diaphragm bending and volume compression
Source: Microsyst Nanoeng. 2024 Mar 15;10:38. doi: 10.1038/s41378-024-00667-8 (PMC10940606; doi:10.1038/s41378-024-00667-8)
Supplement: Supplementary file 1 — Supplemental Material [file 41378_2024_667_MOESM1_ESM.docx]

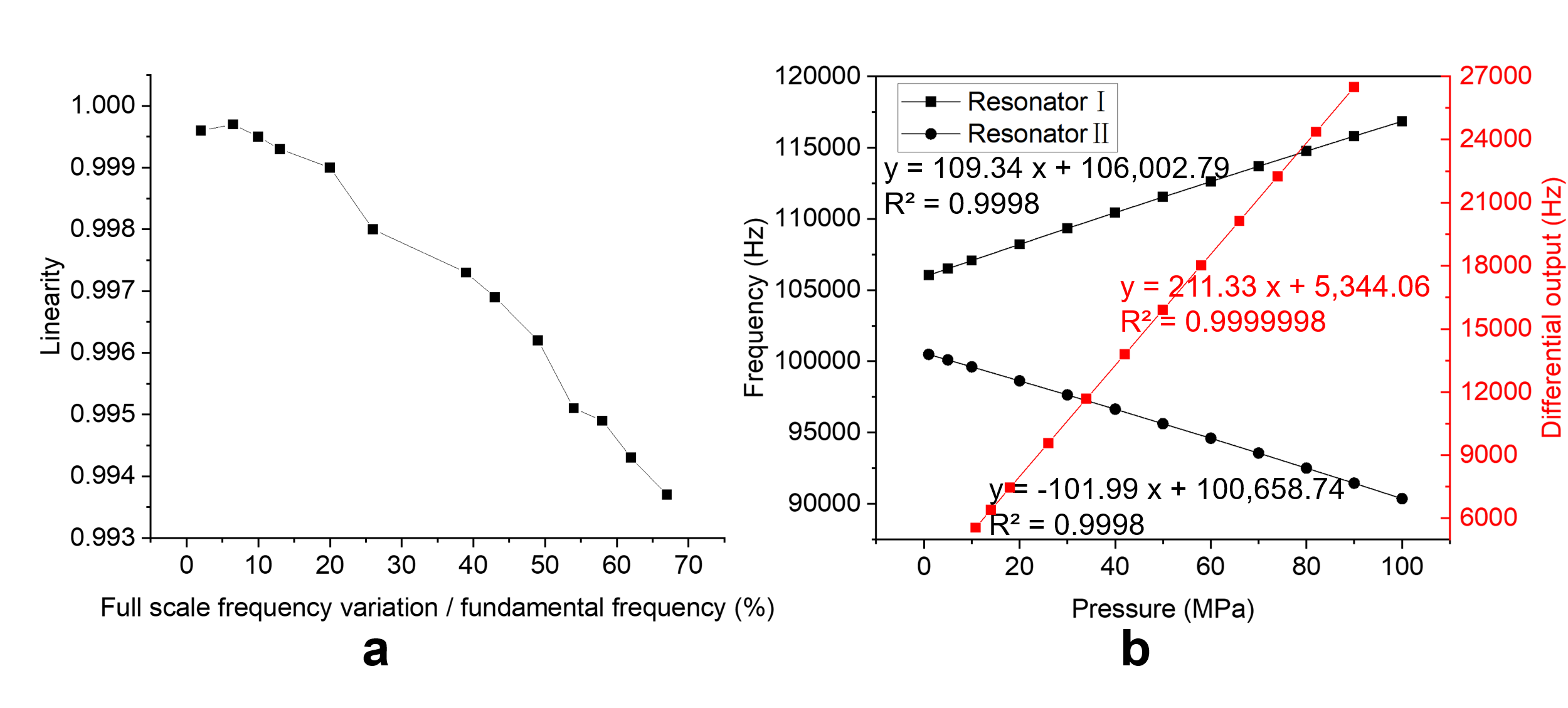


**Fig.S1 a** Linearity at different full scale frequency variations, **b** pressure sensitivities and linearities of dual resonators

The Fig.S1a above shows the relationship between linearity and the full scale frequency variation analyzed by simulation . When the frequency variation is about 10% of fundamental frequency, excellent linearity and sensitivity can be both achieved.

We adopted dual resonators to realize temperature self-compensating, who had full scale frequency variations of ±10% fundamental frequencies respectively. The Fig.S1b shows the pressure sensitivity of dual resonators. Sensitivity and the linearity of the resonator Ⅰ are quantified as 109.34Hz/MPa(~1031.5ppm/MPa) and 0.9998, while sensitivity and the linearity of the resonator Ⅱ are quantified as -101.99Hz/MPa(~-1013.3ppm/MPa) and 0.9998. Differential output can realize higher sensitivity(~2044.8ppm/MPa) and linearity(0.9999998).


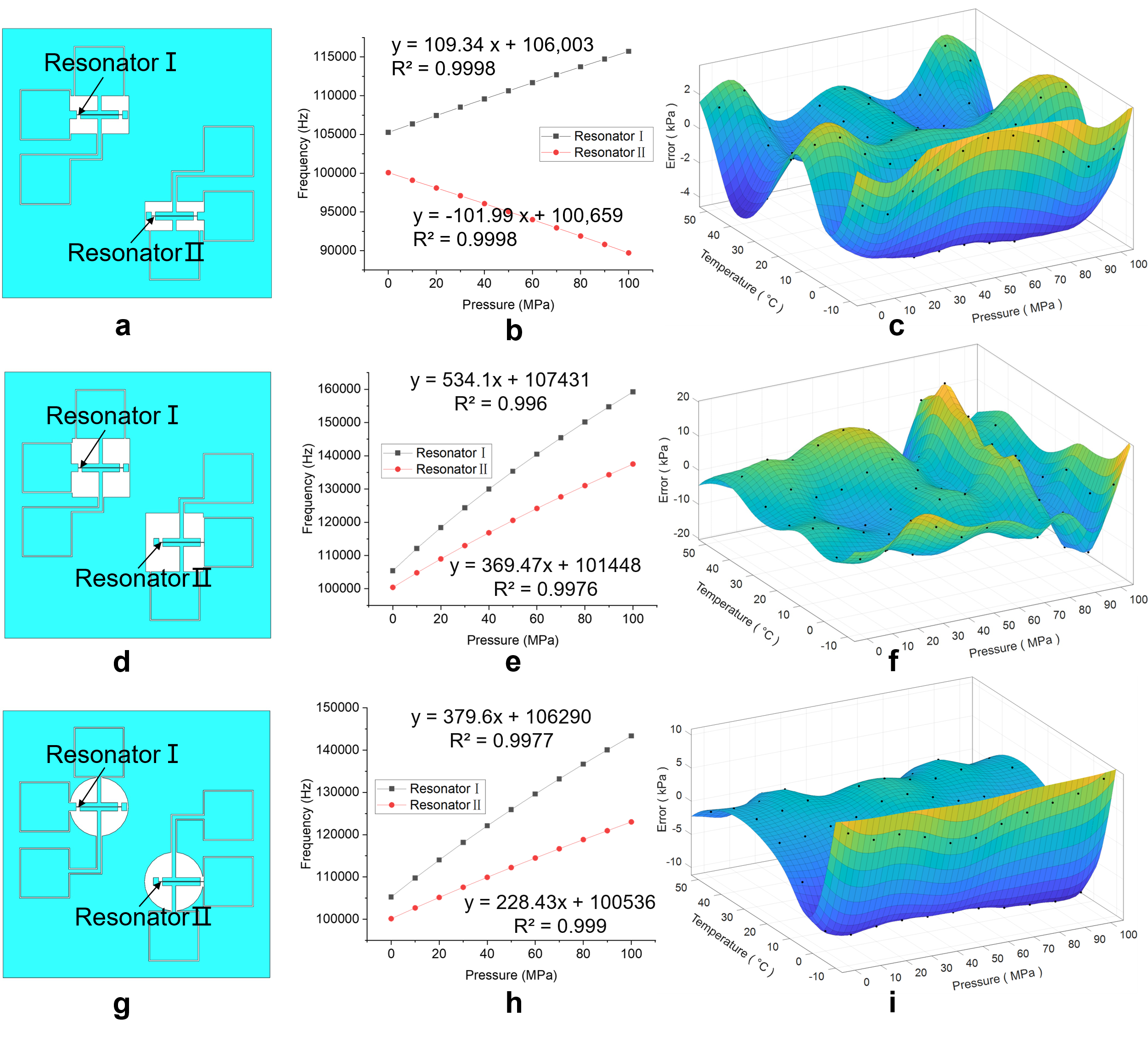


**Fig.S2 a** device layer of rectangular diaphragm microsensor, **b** pressure sensitivities of rectangular diaphragm microsensor, **c** full range fitting errors of rectangular diaphragm microsensor, **d** device layer of square diaphragm microsensor, **e** pressure sensitivities of square diaphragm microsensor, **f** full range fitting errors of square diaphragm microsensor, **g** device layer of circular diaphragm microsensor, **h** pressure sensitivities of circular diaphragm microsensor, **i** full range fitting errors of circular diaphragm microsensor

When the resonant beam is placed in the center of the rectangular diaphragm as shown in Fig.S2a, the stress state on the beam changes gently with the width of the diaphragm, which has excellent stress controllability and process tolerance. It is easy to achieve axially equal tensile and compressive resultant axial stresses on the dual resonators, with a full scale frequency variation of ±10% of the fundamental frequency, performing excellent sensitivity and linearity as shown in Fig.S2b. Then, the frequency of the dual resonators under different temperatures and pressures is obtained through simulation. In a pressure range of 0.1~100 MPa and a temperature range of -10~50℃, Fig.S2c shows the maximum fitting error of the sensor is -4.19kPa and the accuracy is better than ±0.005%FS.

When the diaphragm becomes square or circular under the other conditions(sensor size, strength, cost, fabrication, etc.) are similar with the rectangular. To maintain high pressure resistance, the minimum size of square/circular diaphragm is 1100μm side length / 1100μm diameter. Since the diaphragm size can't be changed, in order to achieve the design of dual resonators to achieve self-compensation, we can only place two resonators in different positions of the miniaturized square/circular diaphragm, because the tensile stress caused by the bending of the diaphragm increases from the edge to the center. However, due to the relatively large area of the square/circular diaphragm, the tensile stress caused by the bending of the diaphragm is always greater than compressive stress, so the two resonators can only achieve positive pressure sensitivity. To achieve differential sensitivity comparable to that of rectangular diaphragm microsensor, the two resonators are placed in the very center and the very edge of the square/circular diaphragm respectively, as shown in Fig.S2d/g. The pressure sensitivity of dual resonators on square/circular diaphragm is shown in Fig.S2e/h, and the full scale variation of frequency is greater than 10% of the fundamental frequency, performing poor linearity. Through simulation and fitting, it can be obtained that the microsensor with square diaphragm has a maximum error of 18.50kPa and an accuracy of ±0.02%FS at full scale, as shown in Fig.S2f. The microsensor with a circular diaphragm has a maximum error of 10.15kPa and an accuracy of ±0.015%FS, as shown in Fig.S2i. So rectangular diaphragm is more conducive to achieve high accuracy measurements in this resonant high-pressure microsensor. In addition , the processes are likely to have greatly different effects on the dual resonators in different positions of the diaphragms in the actual fabrication.
